# Identification of latent classes in mood and anxiety disorders and their transitions over time: a follow-up study in the adult general population

**DOI:** 10.1017/S0033291724001740

**Published:** 2024-09

**Authors:** Margreet ten Have, Marlous Tuithof, Saskia van Dorsselaer, Neeltje M. Batelaan, Brenda W.J.H. Penninx, Annemarie I. Luik, Jeroen K. Vermunt

**Affiliations:** 1 Trimbos Institute, Netherlands Institute of Mental Health and Addiction, Utrecht, the Netherlands; 2 Department of Psychiatry, Amsterdam UMC, Vrije Universiteit Amsterdam, Amsterdam, The Netherlands; 3 Amsterdam Public Health Research Institute, Amsterdam, the Netherlands; 4 Department of Epidemiology, Erasmus MC University Medical Center, Rotterdam, The Netherlands; 5 Department of Methodology and Statistics, Tilburg University, Tilburg, the Netherlands

**Keywords:** anxiety disorders, disease trajectories, epidemiology, latent classes, latent transitions, mood disorders, population surveys, prospective studies

## Abstract

**Background:**

Mood and anxiety disorders are heterogeneous conditions with variable course. Knowledge on latent classes and transitions between these classes over time based on longitudinal disorder status information provides insight into clustering of meaningful groups with different disease prognosis.

**Methods:**

Data of all four waves of the Netherlands Mental Health Survey and Incidence Study-2 were used, a representative population-based study of adults (mean duration between two successive waves = 3 years; N at T0 = 6646; T1 = 5303; T2 = 4618; T3 = 4007; this results in a total number of data points: 20 574). Presence of eight mood and anxiety DSM-IV disorders was assessed with the Composite International Diagnostic Interview. Latent class analysis and latent Markov modelling were used.

**Results:**

The best fitting model identified four classes: a healthy class (prevalence: 94.1%), depressed-worried class (3.6%; moderate-to-high proportions of mood disorders and generalized anxiety disorder (GAD)), fear class (1.8%; moderate-to-high proportions of panic and phobia disorders) and high comorbidity class (0.6%). In longitudinal analyses over a three-year period, the minority of those in the depressed-worried and high comorbidity class persisted in their class over time (36.5% and 38.4%, respectively), whereas the majority in the fear class did (67.3%). Suggestive of recovery is switching to the healthy class, this was 39.7% in the depressed-worried class, 12.5% in the fear class and 7.0% in the high comorbidity class.

**Conclusions:**

People with panic or phobia disorders have a considerably more persistent and chronic disease course than those with depressive disorders including GAD. Consequently, they could especially benefit from longer-term monitoring and disease management.

## Introduction

Researchers have long recognized that mood and anxiety disorder are not isolated disorders, they often co-occur (Kessler et al., 1996; Merikangas et al., 1996), share risk factors (Blanco et al., 2014; Mathew, Pettit, Lewinsohn, Seeley, & Roberts, 2011), and could be seen as a manifestation of the same underlying higher-order (i.e. internalizing) construct (Caspi et al., 2014; Krueger, 1999; Vollebergh et al., 2001).

However, when analyzing these disorders, researchers often have focused on analyzing atypical samples (e.g. pure cases) or have disregarded comorbidity between disorders (Brown, Campbell, Lehman, Grisham, & Mancill, 2001; Jacobi et al., 2004; Kessler, Chiu, Demler, Merikangas, & Walters, 2005). Moreover, all anxiety disorders are often defined as one group and compared to depressive disorders, while there is controversy surrounding the diagnostic categorization of generalized anxiety disorder (GAD). Several studies link GAD more closely to depressive disorders than to other anxiety disorders (Kendler, Prescott, Myers, & Neale, 2003; Kessler et al., 2005; Krueger, 1999; Krueger & Markon, 2006; Slade & Watson, 2006; Vollebergh et al., 2001), while other studies dispute this view (Beesdo, Pine, Lieb, & Wittchen, 2010; Kessler et al., 2008). A data-driven longitudinal study will help inform us how we could cluster people with these disorders most adequately.

Additionally, a growing body of research has found evidence for quite high transition rates over time between depressive and anxiety disorders (Fichter, Quadflieg, Fischer, & Kohlboeck, 2010; Merikangas et al., 2003; Tyrer, Seivewright, & Johnson, 2004). However, few studies examining the longitudinal course of mood and anxiety disorders have incorporated these transitions between disorders. Studies that took co-occurrence and the development of psychopathology beyond the index disorder into account found less favorable disease prognoses. For example, Scholten et al. (2016) showed that the recurrence rate in patients with a pure anxiety disorder more than doubled at 4-year follow-up, from 23.8% to 54.8%, when not only the development of anxiety disorder but also the development of newly arisen depressive disorders were included. In patients with a pure depressive disorder the recurrence rate increased somewhat less steep from 37.6% to 49.7%, when anxiety disorders were also included. Verduijn et al. (2017) showed that the percentage of patients with MDD with a consistently chronic course since baseline (i.e. experiencing a 2-year chronic episode at three successive follow-up assessments) more than tripled at 6-year follow-up, from 4.6% to 14.7%, when a broader definition of disease outcome was applied (from MDD only to including dysthymia, (hypo)mania and anxiety disorders). Similar results have been found in a population-based cohort study by Ten Have et al. (2022). They showed that at 9-year follow-up, the rates of a persistent disorder (a disorder at each follow-up assessment) tripled when besides the index disorder closely related mental disorders were included as relevant disease outcome (MDD: from 4.8% to 13.9%; anxiety disorder: from 4.5% to 15.5%). Yet, it remains unanswered how disease prognosis is when data-supported categorizations of these diagnoses are used. Person-centered latent variable approaches can help identify meaningful classes within a heterogeneous population and visualize the transitions between these classes over time.

To the best of our knowledge, no study has yet examined which latent classes exist in the general population based on the occurrence of both mood and anxiety disorders, and how transitions between these classes are over time. The studies that have been done have examined latent trajectories based on depressive or anxiety symptom scores over time between - by researchers-defined - clinical groups at baseline. Within one large clinical cohort study (Netherlands Study of Depression and Anxiety), the symptom course trajectories differed mainly in severity and/or duration of symptoms when studied separately for patients with a current depressive or anxiety disorder at baseline (Rhebergen et al., 2012; Spinhoven et al., 2016). In line with these studies, Solis et al. (2021) found three latent course trajectories over a nine-year period, varying from chronic, partial recovered and recovered patients, based on mean severity scores of depressive, anxiety, fear, and worry symptoms during follow-up among patients with a current depressive and/or anxiety disorder at baseline. Only one study estimated latent trajectories based on clinical diagnoses among the general population over time (Paksarian et al., 2016). Three latent course trajectories over three decades were found varying from low, increasing-decreasing and increasing disorder levels, but as all disorders were combined into one category it does not yield insight into comorbidity trajectories within and between mood and anxiety disorders.

This study attempts to fill this research gap by analyzing data from the Netherlands Mental Health Survey and Incidence Study-2 (NEMESIS-2), a nationally representative cohort study among adults. We used latent class analysis (LCA) and latent Markov modelling (LMM) to (i) identify latent classes in the general adult population based on the presence of eight different mood and anxiety disorders, and (ii) examine transitions of these latent classes over time. Moreover, to facilitate understanding and detection of the different classes and transitions, we characterized the latent classes using a series of background characteristics and examined predictors of different course transitions. Based on previous studies (Penninx et al., 2011; Scholten et al., 2023; Schopman, Ten Have, Van Balkom, De Graaf, & Batelaan, 2021; Ten Have et al., 2018), we expect indicators of vulnerability and poor health to be predictors of unfavorable latent course transitions.

## Methods

### Study design

NEMESIS-2 is a population-based cohort study with the aim to investigate prevalence and course of common mental disorders in the Dutch general population aged 18–64. It is based on a multistage, stratified random sampling of households, with one respondent randomly selected from each household. Each respondent was interviewed face-to-face. In the first wave (T_0_), performed from November 2007 to July 2009, 6646 individuals were interviewed (response rate 65.1%; average duration: 95 min). The sample was broadly nationally representative, although younger subjects and people of non-Dutch origin were somewhat underrepresented (De Graaf, Ten Have, & Van Dorsselaer, 2010).

All respondents were approached for follow-up, three years (T_1_: *n* = 5303; response rate 80.4%, with those deceased excluded; duration: 84 min), six years (T_2_: *n* = 4618; response rate compared to T_1_ 87.8%; duration: 83 min) and nine years (T_3_: *n* = 4007; response rate compared to T_2_ 87.7%; duration: 101 min) after baseline. Attrition between T_0_ and T_3_ was not significantly associated with any of the assessed 12-month mental disorders at T_0_ after controlling for sociodemographic characteristics (De Graaf, Van Dorsselaer, Tuithof, & Ten Have, 2018). Attrition during follow-up was related to younger age, lower education, unemployment, and non-Dutch origin (De Graaf, Van Dorsselaer, Tuithof, & Ten Have, 2013).

The study was approved by a medical ethics committee (the Medical Ethics Review Committee for Institutions on Mental Health Care, METIGG). After receiving information about the study aims, respondents provided written informed consent at each wave. A comprehensive description of the design can be found elsewhere (De Graaf et al., 2010).

### Measurements

#### Mental disorders

The Composite International Diagnostic Interview (CIDI) version 3.0 was used at all waves to assess mood, anxiety, and substance use disorders according to DSM-IV criteria. The CIDI 3.0 is a fully structured lay-administered interview developed by the World Health Organization, which is used worldwide (Kessler & Üstün, 2004). Clinical reappraisal interviews showed that it has generally good validity for assessing common mental disorders (Haro et al., 2006).

At baseline (T_0_) a lifetime CIDI-version was used; at follow-up (T_1−_T_3_) a CIDI-version with as timeframe the period between the previous and the current wave.

For this paper, mood (major depression, dysthymia, bipolar disorder) and anxiety disorders (panic disorder, agoraphobia, social phobia, specific phobia, GAD) in the past 12 months assessed at each wave, were used. Diagnoses were made without the imposition of hierarchical exclusion rules to facilitate the examination of comorbidity, following Vollebergh et al. (2001) and Caspi et al. (2014).

#### Background characteristics

Sociodemographic, vulnerability, and physical health characteristics were self-reported during the interview and assessed at each wave, unless explicitly stated.

The sociodemographic characteristics used were: sex, age, educational level (only assessed at T_0_ and T_3_; T_0_ information was also used for educational level at T_1_ and T_2_), living without a partner, and having no paid job.

As vulnerability characteristics were used: childhood abuse (whether before age 16 one had experienced emotional neglect, psychological abuse or physical abuse on ⩾2 occasions, or sexual abuse on ⩾1 occasion; only assessed at T_0_; T_0_ information was used at all follow-up waves), and negative live events, which indicated how many of 10 negative life events were experienced in the past 12 months, such as divorce, unemployment and serious financial difficulties, based on Brugha, Bebbington, Tennant, and Hurry (1985).

The physical health characteristics used were: chronic physical disorder (presence of ⩾1 of 17 chronic physical disorders treated or monitored by a medical doctor in the past 12 months, assessed with a standard checklist), body mass index (BMI; kg/m2), excessive drinking (defined as >14/21 drinks weekly for women/men, based on two CIDI questions focusing on the past 12 months: ‘How often did you usually have at least 1 drink?’ and ‘On the days you drank, about how many drinks did you usually have per day?’), smoking (in the past month), and physical exercise (defined as weekly ⩾1 h of physical exercise/sport in the past 12 months; assessed at T_1_, T_2,_ and T_3_, missing at T_0_).

### Statistical analysis

To identify different classes in the study population with respect to their mental health status, we used a 2-step analysis in the statistical program Latent GOLD 6.1 (Vermunt & Magidson, 2021). In this analysis data of all four waves were used.

First, latent class models were built based on the eight mood and anxiety disorders in the pooled dataset (Collins & Lanza, 2009; Magidson & Vermunt, 2004). Models with a different number of latent classes were explored. In the LCA, each wave is considered as a case. There were no missing values on the class indicators, implying the total number of cases in this part of analysis is 20 574. To decrease the likelihood of obtaining local maximum solutions, the number of random start-sets and initial iterations per start-set was increased from the default values of 16 and 50 to 64 and 250, respectively. The assessment of 12-month disorders slightly differed between baseline (identifying symptoms ever in life and then in the past 12 months) and follow-up (identifying symptoms since the previous interview and then in the past 12 months), resulting in higher prevalence rates of the observed disorders at baseline. To account for this difference in measurement (this measurement non-invariance), a variable indicating baseline wave (1) *v.* follow-up wave (0) was added to the estimated LCA model and specified to have a direct effect on the class indicators (disorders). This way, we allowed the prevalence of disorders within the latent classes to be different for the baseline wave compared to the follow-up waves, and prevented changes in the estimated latent classes proportions across waves resulting from the different measurement of the class indicators. We also checked whether the states differed across all waves or time points. This was not the case, as all bivariate residuals between time (coded as 0,1,2,3) and all eight class indicators were well below 1. To determine the optimal number of latent classes, we used the following statistics: three information criteria (IC) including Bayesian IC, Akaike IC and the corrected Akaike IC with a penalty factor of 3 (for all three IC the smallest value is preferred), the likelihood-ratio goodness-of-fit statistics with bootstrap *p* values, the bootstrap likelihood ratio (−2 log-likelihood difference) test, and the bivariate residuals (values smaller than 3 or 4 are preferred). We also took into account the interpretability of the models. Dependency in the observations (multiple observations within one respondent) was checked, but the design effect turned out to be negligible (1.07), meaning that there was no need to correct the statistics for dependent observations in this step.

Second, we investigated the association between the encountered latent classes and the background characteristics described above. Bivariate analyses were performed using the data from all waves (missings excluded), with Wald tests accounting for dependencies between waves to determine significant differences in background characteristics between classes (*p* < 0.05). For this purpose, we used the two-step approach of Bakk and Kuha (2018), which involves estimating a latent class model including background characteristics while fixing the parameters for the class indicators to their estimated values from the first step. We decided to use this approach instead of the more common three-step approach because we obtained several very small latent classes, for which modal class assignment yields huge classification errors. In Latent GOLD, applying the Bakk-Kuha approach involves adding the class-specific log-densities from the first step to the data file and using these in the second step (in a similar manner as one would use the posteriors in a step-three analysis, see: Vermunt, 2010).

Next, we studied transitions across the identified latent classes over time using a latent Markov model (also referred to as latent transition model; Bartolucci, Farcomeni, & Pennoni, 2013; Collins and Lanza, 2009), taking into account the dependency in the data by defining respondent number as primary sampling unit. For this purpose, we also used the two-step approach of Bakk and Kuha (2018), that is, by fixing the model parameters for the class indicators to their estimated values from the first step (Di Mari, Oberski, & Vermunt, 2016). In Markov analysis, each respondent is considered a case. All waves of a respondent for which information is available are included. If a wave is missing, it does not provide information about a transition between two consecutive waves. As a result the total number of cases in these analyses is 6646 and the total number of datapoints included in these analyses is 20 574. The latent Markov model was run multiple times with different background characteristics or covariates (missings imputed with the mean), in order to examine whether the observed transition patterns were affected by these covariates. Because these are in principle exploratory analyses, we looked at one covariate at a time. Due to the large number of tests (12 class transitions for each background characteristic/covariate), we used a stricter significant level (*p* < 0.001). All covariates were time-varying except sex and childhood abuse. The covariates were included at time T to predict latent transitions at time T + 1.

## Results

### Best fitting model

LCA was used to identify latent classes in the general adult population based on the presence of eight different mood and anxiety disorders in the pooled dataset (total number of data points: 20 574). A series of LCA with one-to five-classes was run. The best fitting model, based on the IC-values, the bootstrap *p*-values of the likelihood-ratio goodness-of-fit statistics, the bootstrap likelihood-ratio tests, bivariate residuals and interpretability, was a four-class model (see online supplementary table 1).

### Description of the four latent classes

The first class (prevalence, 94.1%) was labelled ‘healthy’ because it had the lowest probabilities for all mood and anxiety disorders. The second class (prevalence, 3.6%) was characterized by relatively high proportions of adults with major depressive disorder and moderate-to-high proportions with bipolar disorder, dysthymia and GAD and is therefore further referred to as the ‘depressed-worried’ class. The third class (prevalence, 1.8%) is distinguished as ‘fear’ class due to the relatively moderate-to-high proportions of adults with social phobia, specific phobia, agoraphobia, and panic disorder. Compared to the depressed-worried class, the fear class had lower proportions of adults with mood disorder and GAD. The fourth and high comorbidity class (prevalence, 0.6%) had the highest probabilities for almost all mood and anxiety disorders, indicating high comorbidity between disorders.

The four latent classes significantly differed on all background characteristics or covariates of interest (Table 1). Compared to the healthy class, individuals in the three other classes were more often female, younger, lower educated, more often lived without a partner, had no paid job, more often experienced childhood abuse, reported more negative life events, more often had a chronic physical disorder, were excessive drinkers, more often smoked and were less often physical active. Individuals of the high comorbidity class were further characterized by the highest proportion of lower secondary educated, without a partner, without a paid job and smokers; individuals of the fear class by the highest proportion of females, with a history of childhood abuse, negative life events, a chronic physical disorder, a higher BMI-score and of excessive drinkers.

**Table 1.** Sociodemographic and other characteristics for the total population and by latent class, in percentages or means

	Total	Classes					
		1. Healthy	2. Depressed-worried	3. Fear	4. High comorbidity		
	% (SE)	% (SE)	% (SE)	% (SE)	% (SE)		
**Size n (%)**	_	19 354 (94.1)	732 (3.6)	362 (1.8)	126 (0.6)		
**Classes indicators**							
Major depressive disorder	5.5 (0.2)	1.7 (0.3)	89.8 (3.7)	2.1 (6.5)	98.7 (2.6)		
Bipolar disorder	0.6 (0.1)	0.0 (0.0)	12.6 (1.9)	0.0 (0.1)	25.6 (5.4)		
Dysthymia	0.7 (0.1)	0.0 (0.0)	15.8 (2.0)	0.7 (0.9)	22.8 (4.9)		
Generalized anxiety disorder	2.0 (0.1)	0.6 (0.1)	27.8 (3.0)	10.8 (2.7)	50.9 (6.5)		
Social phobia	2.3 (0.1)	1.0 (0.1)	11.4 (2.6)	32.4 (6.0)	71.6 (9.3)		
Specific phobia	3.5 (0.1)	2.3 (0.2)	11.5 (2.1)	40.2 (6.8)	40.1 (6.1)		
Panic disorder	1.2 (0.1)	0.5 (0.1)	9.3 (1.7)	14.4 (3.2)	25.7 (5.2)		
Agoraphobia	0.5 (0.1)	0.0 (0.0)	1.0 (0.9)	11.9 (3.0)	41.1 (9.4)		
	%/mean	%/mean	%/mean	%/mean	%/mean	Wald	P-value
**Sociodemographic**							
Female sex	55.3	54.3	69.0	81.0	59.4	46.6	<0.001
Age (mean (s.d.))	48.5 (12.9)	48.7 (12.9)	45.1 (12.3)	44.3 (12.3)	46.7 (11.2)	37.5	<0.001
Educational level						25.8	<0.001
Lower secondary	30.5	30.1	36.6	34.8	46.2		
Higher secondary	32.0	31.8	33.8	38.0	39.8		
Higher professional, university	37.5	38.1	29.6	27.2	14.0		
Living without a partner	29.2	27.7	53.7	47.2	67.9	152.1	<0.001
No paid job	29.2	28.1	44.5	42.5	61.9	84.9	<0.001
**Vulnerability**							
Childhood abuse	28.6	26.4	58.0	72.9	66.3	225.2	<0.001
Negative life events (mean (s.d.))	0.7 (0.7)	0.6 (0.8)	1.6 (1.3)	3.0 (1.2)	1.3 (1.2)	660.5	<0.001
**Physical health**							
Chronic physical disorder	40.7	39.5	55.7	66.1	63.0	85.8	<0.001
BMI (mean (s.d.))	25.5 (25.6)	25.3 (3.8)	26.0 (5.2)	36.1 (7.8)	25.8 (5.1)	224.7	<0.001
Excessive drinking	5.5	5.2	9.8	15.0	6.8	30.0	<0.001
Smoking	25.7	24.5	42.5	43.5	61.4	99.2	<0.001
Physical exercise	61.3	62.5	41.0	47.3	41.0	69.6	<0.001

*Note 1.* Number of cases on: excessive drinking = 20 539; smoking = 20 431; physical exercise = 13 892; childhood abuse = 20 233; negative life events = 20 434; BMI = 20 510; chronic physical disorder = 20 434. For other characteristics, there are no missing data (i.e. *N* = 20 574 cases).

*Note 2*. The class indicators or disorders occurred in the past 12 months and were assessed according to DSM-IV criteria. The prevalence rates are based on data of all waves.

### Latent transitions of the four latent classes

Table 2 shows the transitions probabilities of these four classes between all two consecutive waves (i.e. from T_0_ to T_1_, T_1_ to T_2_, and from T_2_ to T_3_) taken together, which is a period of three years on average. The mental health of almost all individuals in the healthy class did not change over a three-year period (97.6%) and they remained in this class. Only 1.9% switched to the depressed-worried class, and even lower percentages to the other classes. The mental health of the individuals in the fear class was less stable, but still 67.3% stayed in this class. 14.7% moved to the depressed-worried class, 12.5% to the healthy class and 5.6% transitioned to the high comorbidity class. The majority of individuals in the depressed-worried class and high comorbidity class changed classes over time (63.5% and 61.6%, respectively); over a third remained in their class. The highest percentage in the depressed-worried class moved to the healthy class (39.7%); in the high comorbidity class, it went to the fear class (43.5%). As with the fear class, more than 5% of the depressed-worried class transitioned to the high comorbidity class. Suggestive of recovery is switching to the healthy class, this was 39.7% in the depressed-worried class, 12.5% in the fear class and 7.0% in the high comorbidity class.

**Table 2.** Latent transitions of the identified latent classes between two consecutive waves over time, based on a Markov model with the Bakk-Kuha adjustment method, in percentages

		Class at time T + 1			
Class at time T	Size n (%)	1. Healthy % (SE)	2. Depressed-worried % (SE)	3. Fear % (SE)	4. High comorbidity % (SE)
1. Healthy	19 354 (100%)	97.6 (0.2)	1.9 (0.2)	0.4 (0.1)	0.1 (0.1)
2. Depressed-worried	732 (100%)	39.7 (3.7)	36.5 (3.6)	18.6 (3.4)	5.2 (1.8)
3. Fear	362 (100%)	12.5 (3.4)	14.7 (3.4)	67.3 (4.3)	5.6 (2.1)
4. High comorbidity	126 (100%)	7.0 (6.8)	11.2 (6.7)	43.5 (10.2)	38.4 (8.8)

*Note*. The transition between classes with the smallest number of people was from class 4 to class 1. This involved less than 10 persons (i.e. 126*0,07).

### Predictors of the latent transitions

In subsequent analyses, we investigated predictors of the transitions between latent classes (see Table 3 for a summary and the online supplementary Tables 2a-2d for the statistics). In addition to demographic characteristics, indicators of greater vulnerability or poorer physical health predicted an unfavorable transition. For example, transitions from the healthy class to the depressed-worried class were significantly associated with female sex, younger age, living without a partner, having experienced childhood abuse, more negative life events, chronic physical disorder, and not being physical active; and transitions to the fear class with younger age, living without a partner, more negative life events, excessive drinking, and smoking. Less predictors were found for a favorable transition. For example, transitions from the depressed-worried class to the healthy class were significantly associated with having a paid job and fewer life events; and transitions from the fear class to the healthy class were significantly associated with a lower educational level.

**Table 3.** Predictors of latent transitions

	Class at time T + 1			
Class at time T	1. Healthy	2. Depressed-worried	3. Fear	4. High comorbidity
1. Healthy	−	Female sex Younger age Living without a partner Childhood abuse Negative life events Chronic physical disorder Physical inactive	Younger age Living without a partner Negative life events Excessive drinking Smoking	None
2. Depressed-worried	*Paid job Fewer negative life events*	−	Younger age	None
3. Fear	*Lower educational level*	None	−	None
4. High comorbidity	None	*Lower BMI*	*Fewer negative life events* *Excessive drinking*	−

--: not applicable, this is the reference category for studying transitions between classes.

none: no significant predictors found at *p* < 0.01.

The predictors associated with an improvement in mental health between classes over time are shown in italics.

All predictors were time-varying and assessed at time T in order to predict latent transitions at time T + 1, except for sex and childhood abuse which were not time dependent.

No significant predictors were found of transitions to the high comorbidity class from any other class, nor from the fear class to the depressed-worried class, and nor from the high comorbidity class to the healthy class.

## Discussion

### Key findings

This study showed the existence of four different latent classes in the general population based on the occurrence of eight mood and anxiety disorders; a healthy class, depressed-worried class, fear class and high comorbidity class. These classes not only differed in prevalence, baseline characteristics but also in disease transitions. Suggestive of recovery is switching to the healthy class over a three-year period, this was only 39.7% in the depressed-worried class, 12.5% in the fear class and 7.0% in the high comorbidity class. These findings show the chronicity of mood and especially anxiety disorders and stress the need to provide regular monitoring and disease management.

### Strengths and limitations

Our population-based study has important strengths, including the large sample of adults, the prospective design, and the use of a diagnostic instrument to assess mood and anxiety disorders at each wave. Yet, some limitations deserve discussion. First, although the sample was representative of the Dutch population on most parameters, people with an insufficient mastery of Dutch, those with no permanent residential address and the institutionalized were underrepresented. Hence, our findings cannot be generalized to these groups, such as the most severely affected depressed and anxious patients. Second, despite the fact that the analyses were based on the full NEMESIS-2 dataset with a total of 20 574 data points, the number of individuals in the three mentally unhealthy classes that switched to another class was not always large enough to study robust predictors of these transitions. This means that non-significant predictors of these transitions can become significant with sufficient power. Third, the latent classes and their transitions over time were based on the presence of mood and anxiety disorders. Disorders can vary in the impairments people experience from them in their daily life. This means that a persistent or chronic disease course not always is the most serious course; after all, a short illness duration can be accompanied by severe role limitations.

### Discussion of research findings

This study identified four latent classes in the general adult population based on the presence of eight mood and anxiety disorders: a healthy class (prevalence: 94.1%), depressed-worried class (3.6%; moderate-to-high proportions with all mood disorders and GAD), fear class (1.8%; moderate-to-high proportions with panic disorder and all phobia disorders) and high comorbidity class (0.6%). The prevalence of these latent classes did not differ between the measurement waves, indicating robust classes over time.

The present study found that the prevalence of GAD was lower in the anxiety class compared to the depressed worried class and the high comorbidity class. This is in line with several previous studies (Kendler et al., 2003; Kessler et al., 2005; Krueger, 1999; Krueger & Markon, 2006; Slade & Watson, 2006; Vollebergh et al., 2001) that have suggested a closer link of GAD to depressive disorders than to other anxiety disorders. Other studies (Beesdo et al., 2010; Kessler et al., 2008) dispute this view although they did not rely on latent variable approaches. More work is needed to determine whether a distinction between panic disorders and phobias on the one hand and mood disorders including GAD on the other hand is helpful in future research when assessing mechanisms, course and treatment.

Compared to all other latent classes, individuals in the fear class were characterized by the highest proportion of females, with a history of childhood abuse, negative life events, a chronic physical disorder, a higher BMI-score and of excessive drinkers. They also had higher percentages of physically active individuals and those living with a partner compared to those in the depressed-worried class. That these two latent classes differed with respect to baseline demographic, vulnerability and physical health characteristics adds to previous findings that both classes are different manifestations of the same underlying higher-order (i.e. internalizing) disease construct (e.g. Vollebergh et al., 2001).

Individuals of the high comorbidity class were characterized by the highest proportion of smokers and those with a lack of resources (lower educated, living without a partner, unemployed). Although these adverse economic and social conditions did not play a role in the transition to a healthier class, these factors may be particularly important to consider when treating the most affected patients.

Persistency (i.e. remaining in the same class over time) and chronicity (i.e. staying in one of the classes with moderate-to-high proportions of disorders over time) were different between the latent classes over time. We found that people in the fear class had a more persistent and chronic disease course than those in the depressed-worried class. This contrasts with previous clinical and population-based studies that show better or similar course trajectories of anxiety disorders (often including GAD but excluding specific phobia) compared to depressive disorders (Penninx et al., 2011; Ten Have et al., 2022). An explanation for this may be the composition of the groups as one large clinical cohort study found that remittance rates were more favorable for GAD than for phobias (Hendriks, Spijker, Licht, Beekman, & Penninx, 2013). Additionally, several other psychiatric epidemiological studies found that specific phobias often have a more persistent course than other anxiety disorders such as GAD, based on the 12-month to lifetime prevalence ratios for anxiety disorders (De Graaf, Ten Have, Van Gool, & Van Dorsselaer, 2012; Kessler, Ruscio, Shear, & Wittchen, 2010; Kringlen, Torgersen, & Cramer, 2001). A possible explanation for this is that people with specific phobia are less often impaired by their disorder (Kessler et al., 2005; Ten Have, Nuyen, Beekman, & De Graaf, 2013b), and are less likely to seek professional help (Ten Have, De Graaf, Van Dorsselaer, & Beekman, 2013a; Wang et al., 2005), causing the symptoms to persist.

The finding that the majority of the people in the classes with moderate-to-high proportions of disorders did not switch to the healthy class over a three-year period, shows the chronicity of mood and anxiety disorders. Outpatient mental health services generally focuses on treating the mental disorder and less on preventing relapse and chronicity of the disorder (Hermens, Muntingh, Franx, Van Splunteren, & Nuyen, 2014; Roy-Byrne, Wagner, & Schraufnagel, 2005). The present findings have implications for the way mental health care ideally is organized and the extent regular monitoring, relapse prevention, easy access to consultation, rapidly scaling up care when needed, and maintenance treatment are provided. Clinicians should consider interventions aimed at treating mood and anxiety disorder as chronic and non-isolated disorders by more systematically incorporating regular monitoring and relapse prevention strategies, such as increasing patients’ awareness of signs of relapse, making a relapse prevention plan and agreeing on the steps to be taken in case of recurrence. Besides, treatment programs for chronic or treatment-resistant outpatients should be implemented, and disease management may be better adapted to serve patients with chronic course trajectories.

On the other hand, based on previous population-based studies reporting a treatment gap or delays in help-seeking behavior (Iza et al., 2013; Mekonen, Chan, Connor, Hides, & Leung, 2021; Ten Have et al., 2013b; 2022; Wang et al., 2005), a fairly large proportion of the individuals in the classes with moderate-to-high proportions of disorders might not have sought professional help for their mental disorder. For these people, public awareness on the benefits of timely treatment for mood and anxiety disorder should be raised.

In addition to sociodemographic characteristics, indicators of vulnerability and poor health were found to be predictors of unfavorable latent course transitions. We found more predictors for developing mood and/or anxiety disorders than for its course (i.e. persistence or chronicity of disorders). For example, female sex played a role in the transition from the healthy class to the depressed-worried class, but not the other way around. This finding emphasizes that predictors for disease onset and course may differ.

In conclusion, we found four latent classes: a healthy class, depressed-worried class (mood disorders including GAD), fear class (panic disorder and all phobia disorders) and high comorbidity class. The fear class was more persistent and chronic in nature compared to the depressed-worried class. Our findings suggest that anxiety and depression should be considered jointly in research and clinical practice, particularly as a substantial percentage of people transfers from depressed-worried to fear or vice versa, suggesting that both chronicity and persistency should be taken into account. Moreover, combining the anxiety disorders into one group seems insufficient, as these disorders may group beyond the category of anxiety disorders. This in turn may hamper our understanding of these diseased cases when it comes to their course and effects of treatment.

## Supporting information

ten Have et al. supplementary material ten Have et al. supplementary material

## Supplementary material

The supplementary material for this article can be found at https://doi.org/10.1017/S0033291724001740.
