# Supplementary material for: Identification of latent classes in mood and anxiety disorders and their transitions over time: a follow-up study in the adult general population
Source: Psychol Med. 2024 Sep 26;54(12):3417–24. doi: 10.1017/S0033291724001740 (PMC11496236; doi:10.1017/S0033291724001740)
Supplement: ten Have et al. supplementary material [file S0033291724001740sup001.docx]

Supplementary Table 1 Fit statistics for different Latent Class Models.

| Model | LL | BIC | AIC | AIC3 | NPar | L^2^ | Bootstrap p | -2 LL Diff | Bootstrap p | Max BVR | Sample size per state^a^ |
| --- | --- | --- | --- | --- | --- | --- | --- | --- | --- | --- | --- |
| 1 class | -15356.28 | 30871.47 | 30744.56 | 30760.56 | 16 | 4284.85 | 0.000 |  |  | 2440.96 | 100.0 |
| 2 classes | -13483.77 | 27215.83 | 27017.53 | 27042.53 | 25 | 539.83 | 0.000 | 3745.03 | 0.000 | 38.69 | 95.6 / 4.4 |
| 3 classes | -13402.24 | 27142.17 | 26872.49 | 26906.49 | 34 | 376.78 | 0.000 | 163.05 | 0.000 | 23.74 | 94.8 / 3.5 / 1.8 |
| 4 classes | -13351.97 | 27131.00 | 26789.94 | 26832.94 | 43 | 276.23 | 0.616 | 100.55 | 0.000 | 1.62 | 94.1 / 3.6 / 1.8 / 0.6 |
| 5 classes | -13344.54 | 27205.53 | 26793.07 | 26845.07 | 52 | 261.37 | 0.630 | 14.86 | 0.14 | 2.18 | 94.3 / 3.3 / 0.9 / 0.9 / 0.6 |

*Note*. LL = Log likelihood; BIC = Bayesian information criterion; AIC = Akaike information criterion;

AIC3: Corrected AIC with a penalty factor of 3; NPar = Number of parameters; L^2^: L-squared; -2 LL Diff: minus twice Log likelihood difference between two models; BVR: bivariate residual. In bold = best fitting model overall

^a^ Percentage of total sample size

Supplementary Table 2a Predictors of latent transitions from the healthy class to other classes:

|  | Healthy to Depressed-worried | | | Healthy to Fear | | |
| --- | --- | --- | --- | --- | --- | --- |
| predictor | coefficient | s.e. | p-value | coefficient | s.e. | p-value |
| Female sex | 0.83 | 0.21 | <0.001 |  |  | n.s. |
| Age | -0.03 | 0.01 | <0.001 | -0.07 | 0.02 | 0.002 |
| Living without a partner | 0.86 | 0.18 | <0.001 | 1.83 | 0.57 | 0.001 |
| Childhood abuse | 0.89 | 0.19 | <0.001 |  |  | n.s. |
| Negative life events | 0.69 | 0.06 | <0.001 | 0.93 | 0.17 | <0.001 |
| Chronic physical disorder | 0.50 | 0.18 | 0.006 |  |  | n.s. |
| Physical active | -0.95 | 0.19 | <0.001 |  |  | n.s. |
| Excessive drinking |  |  | n.s. | 1.78 | 0.66 | 0.007 |
| Smoking |  |  | n.s. | 1.68 | 0.63 | 0.008 |

n.s.: not significant at p<0.01

Supplementary Table 2b Predictors of latent transitions from the depressed-worried class to other classes:

|  | Depressed-worried to Healthy class | | | Depressed-worried to Fear | | |
| --- | --- | --- | --- | --- | --- | --- |
| predictor | coefficient | s.e. | p-value | coefficient | s.e. | p-value |
| Age |  |  | n.s. | -0.05 | 0.02 | 0.005 |
| No paid job | -0.91 | 0.30 | 0.003 |  |  | n.s. |
| Negative life events | -0.64 | 0.18 | <0.001 |  |  | n.s. |

n.s.: not significant at p<0.01

Supplementary Table 2c Predictors of latent transitions from the fear class to other classes:

|  | Fear to Healthy | | |
| --- | --- | --- | --- |
| predictor | coefficient | s.e. | p-value |
| Educational level | -4.68 | 1.14 | <0.001 |

Supplementary Table 2d Predictors of latent transitions from the high comorbidity class to other classes:

|  | High comorbidity to Depressed-worried | | | High comorbidity to Fear | | |
| --- | --- | --- | --- | --- | --- | --- |
| predictor | coefficient | s.e. | p-value | coefficient | s.e. | p-value |
| Negative life events |  |  | n.s. | -0.79 | 0.28 | 0.005 |
| BMI | -0.34 | 0.13 | 0.007 |  |  | n.s. |
| Excessive drinking |  |  | n.s. | 5.22 | 1.68 | 0.002 |

n.s.: not significant at p<0.01
